# 上海市肺科医院磨玻璃结节早期肺腺癌的诊疗共识（第一版）

**DOI:** 10.3779/j.issn.1009-3419.2018.03.05

**Published:** 2018-03-20

**Authors:** 格宁 姜, 昶 陈, 余明 朱, 冬 谢, 洁 戴, 凯淇 靳, 莹冉 沈, 海峰 王, 辉 李, 兰军 张, 树庚 高, 克能 陈, 雷 张, 晓 周, 景云 史, 浩 汪, 博雄 谢, 雷 蒋, 江 范, 德平 赵, 乾坤 陈, 亮 段, 文新 何, 逸鸣 周, 鸿程 刘, 晓刚 赵, 鹏 张, 雄 秦

**Affiliations:** 1 200043 上海，同济大学附属上海市肺科医院 Tongji University afliated Shanghai Pulmonary Hospital, Shanghai 200043, China; 2 100020 北京，首都医科大学附属 北京朝阳医院 Capital Medical University Afliated Beijing Chao-Yang Hospital, Beijing 100020, China; 3 510060 广州，中山大学附属肿瘤医院 Sun Yat-sen University Cancer Center, Guangzhou 510060, China; 4 100021 北京，国家癌症中心/中国医学科学院北京协和医学院肿瘤医院 National Cancer Center/Cancer Hospital, Chinese Academy of Medical Science and Peking Union Medical College, Beijing 100021, China; 5 100142 北京，北京大学肿瘤医院 Peking University Cancer Hospital and Institute, Beijing 100142, China

**Keywords:** 肺磨玻璃结节, 原位腺癌, 微浸润腺癌, 浸润性腺癌, 外科, Pulmonary ground-glass nodules, Adenocarcinoma in situ, Minimal invasive adenocarcinoma, Invasive adenocarcinoma, Surgery

## Abstract

随着胸部计算机断层扫描（computed tomography, CT）检查，尤其是低剂量薄层CT筛查项目在中国的广泛开展，越来越多的无症状肺部磨玻璃结节（ground-glass nodules, GGNs）被发现。虽然国内及国际上已发布了一系列针对肺部GGNs的指南，但是这些指南的撰写者多来自呼吸、肿瘤及影像专业，可能缺乏对现代微创胸外科的充分认识，造成外科手术在肺部GGNs诊治中的作用不明确，甚至被低估；而且，肺部肿瘤相关的各学科对于早期肺癌，尤其是浸润前病变的处理也缺乏统一规范。因此，基于国内外现有文献及上海市肺科医院多年积累的经验，上海市肺科医院撰写了此诊疗共识。本共识推荐对于疑似肺腺癌的GGNs进行多学科评估，依据诊断，选择合理的处置方式。对于疑似原位腺癌，推荐进行胸部薄层CT随访，或在特定情况下进行不超过肺段切除的限制性肺切除；对于疑似微浸润腺癌，推荐进行限制性肺切除或肺叶切除；对于疑似浸润性腺癌，建议依据病灶是否含有磨玻璃成分、位置、大小、个数及患者躯体情况选择合理的手术方式；而肺多发结节的处理原则推荐为主病灶优先，兼顾次要病灶，综合选择治疗方案。

## Abstract (en)

As computed tomography (CT) screening for lung cancer becomes more common in China, so too does detection of pulmonary ground-glass nodules (GGNs). Although anumber of national or international guidelines about pulmonary GGNs have been published, most of these guidelines are produced by respiratory, oncology or radiology physicians, who might not fully understand the progress of modern minimal invasive thoracic surgery, and these current guidelines may overlook or underestimate the value of thoracic surgery in the management of pulmonary GGNs. In addition, the management for pre-invasive adenocarcinoma is still controversial. Based onthe available literature and experience from Shanghai Pulmonary Hospital, we composed this consensus about diagnosis and treatment of pulmonary GGNs. For lesions which are considered as adenocarcinoma *in situ*, chest thin layer CT scan follow-up is recommended and resection can only be adopt in some specifc cases and excision should not exceed single segment resection. For lesions which are considered as minimal invasive adenocarcinoma, limited pulmonary resection or lobectomy is recommended. For lesions which are considered as early stage invasive adenocarcinoma, pulmonary resection is recommend and optimal surgical methods depend on whether ground glass component exist, location, volume and number of the lesions and physical status of patients. Principle of management of multiple pulmonary nodules is that primary lesions should be handled with priority, with secondary lesions taking into account.

## 1 引言

近年来，随着胸部计算机断层扫描(computed tomography, CT)检查，尤其是低剂量薄层CT筛查项目在中国的广泛开展，越来越多的无症状肺部磨玻璃结节(ground glass nodules, GGNs)被发现。其发病特点包括：以东亚裔人群最为常见，非吸烟为主的人群，女性患者，低龄化表现。

目前国内及国际上，发布了一系列针对肺部GGNs的指南，但由于这些指南的撰写者多来自呼吸、肿瘤及影像专业，缺乏对现代微创胸外科的充分认识，因此造成现有指南对外科手术在肺部GGNs诊治中的作用不明确，甚至被低估；而且，肺部肿瘤相关的各学科对于早期肺癌，尤其是浸润前病变的处理也缺乏统一规范。

2011年，肺腺癌新分类^[1]^将肺腺癌分为：浸润前病变、微浸润腺癌(minimally invasive adenocarcinoma, MIA)以及浸润性腺癌。浸润前病变分为不典型腺瘤样增生(atypical adenomatous hyperplasia, AAH)和原位腺癌(adenocarcinoma *in situ*, AIS)。

本共识推荐对疑似肺腺癌的G GNs进行多学科评估，依据诊断，选择合理的处置方式。现对于疑似AIS、MIA、浸润性腺癌及多原发肺癌的处置原则分叙如下。

## 2 原位腺癌

AIS典型的影像学表现为直径大于5 mm且小于30 mm的纯磨玻璃结节(pure ground-glass nodules, pGGN)。pGGN，是指CT肺窗上的局灶性磨玻璃样阴影，且结节内不含能够遮挡血管或支气管结构的实性成分^[2]^。AIS需要与AAH和MIA进行鉴别。小于5 mm的pGGN通常为AAH^[3]^，若pGGN最大径为2 mm-5 mm，其为AAH的可能性约为97%^[4]^；CT值小于-520 HU亦提示AAH的可能^[5]^。若pGGN≥6.5 mm、边界完整^[5]^，或CT上出现血管形态改变^[6]^，或出现空泡征(vacuole)，则AAH的可能较小。AIS还需与MIA鉴别，若GGN出现分叶征，胸膜牵拉^[7]^，支气管充气征^[5]^，通常提示MIA。GGN实性部分平均CT值在鉴别MIA与浸润前病变(A AH/AIS)亦有重要意义，浸润前病变实性成分平均CT值为-318.1 HU，而MIA为-194.7 HU^[8]^。

对首次发现的疑似AIS的GGN应进行定期随访^[9]^。推荐在结节首次发现后的3个月进行首次的薄层CT平扫检查^[2, 10]^；若患者首次CT检查层厚大于3 mm，建议在1个月后复查薄层CT平扫以获得结节的基线资料^[10]^，然后3个月后再次复查薄层CT平扫观察结节的变化情况。

随访过程中，若结节明显缩小，则考虑良性病变可能。若患者年龄小于40岁，无吸烟史及二手烟暴露史，无肺癌家族史，无肺部其他需长期随访的疾病(慢性阻塞性肺病、肺纤维化、支气管扩张等)，则无需常规随访胸部CT；其他患者推荐进行每年1次的薄层CT随访^[11, 12]^。

若结节持续稳定存在，可视病灶形态及大小等因素继续随访。对于直径小于8 mm，CT值较低，边界模糊的pGGN可每半年或1年随访一次；对于直径大于等于8 mm，边界清楚的pGGN，或含有实性成分的部分实性结节(part-solid nodules)，可适当缩短随访时间间隔至6个月；对于持续稳定存在的外周优势部位的疑似AIS病变，也可考虑微创外科手术切除。其依据如下：①部分pGGN仍具有生长和恶变的可能^[13, 14]^；②亚肺叶切除即可将病灶完整切除，且不影响患者预后^[15, 16]^；③术前无需进行正电子发射型断层显像(positron emission tomography, PET-CT)、头颅磁共振、支气管镜等，术前检查简单^[17, 18]^；④术中无需进行纵隔淋巴结清扫/采样，手术创伤小^[15, 19]^；⑤手术可在一定程度上降低患者焦虑水平，改善生活质量^[20]^。外科手术治疗AIS也存在一定的缺点：①若患者手术后再次出现其他部位的肺部结节，可能需要再次手术，则再次手术的难度和风险有可能增加；②AIS进展到危及生命，可能需要多年，过早的手术介入，会导致过早的手术损伤，术后可能出现的长期疼痛或其他并发症，可能影响患者的生活质量，早期手术与随访择期手术相比并不能显著改善患者总体生存，但却可能使患者过早的承受手术的风险及术后疼痛；③术前的AIS的诊断依赖影像学判断，缺乏病理支持，对术前判断的AIS进行手术，术后可能证实为AAH或良性病变。所以，对于稳定存在的AIS应当进行胸部薄层CT随访，仅当同时满足以下4个条件时，可考虑对患者进行外科手术治疗：①对于结节诊断AIS的准确性较高(MDT讨论或结节直径≥8 mm且边界清楚的pGGN)；②结节位于外周或优势段，行楔形切除、亚肺段或肺段切除可完整切除病灶；③随访过程中，患者存在明显的焦虑症状，影响生活质量；④患者无影响其生命的其他系统的严重基础疾病，预期寿命超过10年，且不伴有其他恶性肿瘤。

若结节随访过程中出现体积增大，或实性成分增多，考虑疾病进展为MIA或浸润性腺癌时，应考虑及时外科介入。AIS的处理流程如图 1所示。

**1:**
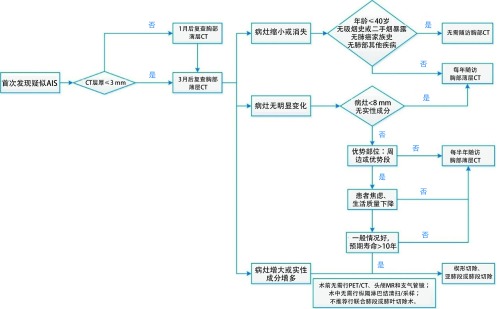
原位腺癌的处理流程图 Management of adenocarcinoma *in situ*

【**肺科共识**——**原位腺癌**】

**术前随访**

可疑AIS患者，术前至少随访1次，且距首次发现间隔3个月以上。

**术前检查**

术前推荐薄层胸部CT平扫，无需行头颅磁共振、全身骨扫描、气管镜、胸部CT增强、PET/CT或经皮肺穿刺检查。

**手术指征**

1.长期随访，结节持续存在；

2.对于结节诊断AIS的准确性较高(MDT讨论或结节直径≥8 mm且边界清楚的pGGN)；

3.结节位于外周或优势段，行楔形切除、亚肺段或肺段切除可完整切除病灶；

4.随访过程中，患者存在明显的焦虑症状，影响生活质量；

5.随访中，结节明显增大或密度变实；

6.患者不伴有影响其生命的其他系统的严重基础疾病，或其他恶性肿瘤，患者的预期寿命超过10年。

**手术原则与手术切除范围**

1.如病灶位于周边"优势部位"：行肺楔形切除；

2.如病灶位置较深，但仍位于某一个肺段内：行亚肺段或肺段切除；

3.病灶处于多个肺段之间或支气管根部，切除需要联合肺段切除或肺叶切除者，不推荐或慎重选择手术；

4.手术切缘应符合基本肿瘤学原则；

5.术中冰冻病理结果决定是否需要扩大切除及淋巴结清扫。

**淋巴结清扫范围**

术中无需淋巴结清扫或采样。

**术后辅助治疗**

术后不需要放疗、化疗或靶向治疗。

**预后**

完全切除后肿瘤学预后良好，5年生存率可达100%。

**术后随访**

如无明显残余病灶，AIS术后可每年复查一次胸部CT平扫，不必复查头颅磁共振、全身骨扫描、气管镜或血肿瘤标志物。

## 3 微浸润腺癌

MIA是一类早期肺腺癌(≤3 cm)，主要以贴壁方式生长，且病灶中任一浸润病变的最大直径≤5 mm，不伴有浸润胸膜、血管、淋巴管或肿瘤性坏死^[1]^。其在影像学上多数表现为pGGN，也有部分表现为部分实性结节，极少数表现为实性结节。在Lee等^[21]^的研究中，三类影像表现的比例分别为53.8%、42.3%和3.8%。在pGGN中，结节的直径及特殊影像征象的有无决定结节的浸润性。持续存在的，直径≥10 mm的pGGN是MIA的一个CT特征^[22]^，其CT值约为(-536.2±113.1) HU^[8]^。分叶征、胸膜牵拉^[7]^和支气管充气征^[5]^的出现可作为MIA与AAH/ AIS鉴别的要点。而对于部分实性结节，根据其内实性成分的比例来区分浸润前病变(AIS/MIA)和浸润性腺癌至关重要。MIA的实性成分最大径一般小于5 mm^[23-26]^，实性面积占比总面积(consolidationtotumor ratio, C/T) < 0.25^[25, 27, 28]^，结节CT值约为(-517.5±161.2) HU，实性部分平均CT值为-194.7 HU^[15]^。对于实性成分的测量目前仍存在较多争议。Fleischner学会推荐在纵隔窗中进行^[3]^，然而也有研究^[29]^指出肺窗上测量得到的实性成分与病理诊断更为符合。故仍需更多的研究来解答这个问题。表现为实性结节的MIA较少见^[15]^。

PET/CT对于pGGN良恶性鉴别及术前分期的价值有限，假阴性率高^[30]^：pGGN的摄取很低或无摄取；部分实性结节大多数呈低摄取(随着实性成分比例增加，摄取值会相应升高)。pGGN很少出现气道内转移或远处转移，传统的气管镜下刷检或穿刺诊断阳性率低，径向探头EBUS下，GGN病变可表现为暴风雪征，活检有利于此类病灶的术前确诊^[31]^。pGGN发生远处转移或气道内转移的风险小，术前无需进一步检查^[17, 18]^，目前有待进一步大规模证据证实。

若影像学上高度怀疑MIA，则需考虑手术切除。术中对于肺部结节的定位方法包括术中肉眼观察、卵圆钳胸膜表面滑动定位、术中手指触诊定位、术中胸腔镜B超探头定位，术中CT定位等。对于直径大、CT值高或靠近胸膜表面或肺裂的结节，多数可通过肉眼观察结合术中触诊定位。术前定位则包括3D重建、CT引导下标记、电磁导航支气管镜引导定位、Virtual Assisted LungMapping(VAL-MAP)等。术前CT引导定位包括：胸膜表面亚甲蓝注射、经皮肺穿刺放置微弹簧圈、经皮肺穿刺Hookwire定位、放射性示踪剂注射、影像辅助导航定位等。

大量研究^[32, 33]^表明，在肿瘤小于2 cm的Ia期患者中，肺叶切除与亚肺叶总体生存无显著差异。影像评估若为MIA，目前的国际共识是局部切除即能达到痊愈。手术方式需遵循个体化原则，综合病灶的具体部位以及患者的身体状况制定最优方案。如病灶位于周边"优势部位"，可行楔形切除；病灶位置较深，但仍位于某一个肺段，可考虑行肺段切除；病灶位置处于两个或多个肺段之间或支气管根部，需行肺叶切除或联合肺段切除。术中冰冻诊断AIS/MIA是可行的。在Liu等^[34]^的研究中，AIS/MIA术中冰冻和术后病理的诊断一致率高达95.9%，术后病理升级为浸润性腺癌的发生率为4.6%，其中贴壁型占56.5%，腺管型占39.1%，乳头型占4.3%。在He等^[35]^的研究中，术中冰冻与术后病理不一致的发生率为4.41%。根据我院经验，若术中行楔形切除，术后病理升级为浸润型腺癌，根据浸润型腺癌亚型决定下一步处理方案：若为微乳头型或实体型，建议再次手术行肺段切除或肺叶切除；若为贴壁型、乳头型或腺管型，建议随访^[36]^。

综上所述，可疑MIA病灶具有手术切除的指征。肺叶切除和亚肺叶切除均可适用于MIA，手术方式的选择取决于病灶的位置和患者的身体状况。完全切除后肿瘤学预后良好，目前文献报道5年生存率可达100%，累积复发率为0%^[37, 38]^，由于样本量较小，仍需进一步临床研究证实。MIA的处理流程如图 2所示。

**2:**
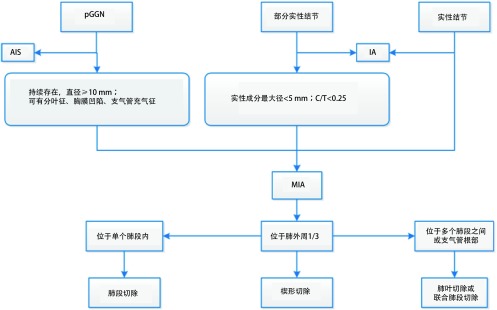
微浸润腺癌的处理流程图 Management of minimal invasive adenocarcinoma

【**肺科共识**——**微浸润腺癌**】

**术前随访**

可疑MIA患者，术前至少随访1次，且距首次发现间隔3个月以上。

**术前检查**

术前推荐薄层胸部CT平扫，而头颅磁共振、全身骨扫描、气管镜、胸部CT增强、PET/CT或经皮肺穿刺等为非必须检查项目，可根据患者具体情况进行选择。

**手术指征**

1.长期随访，结节持续存在；

2.对于结节诊断MIA的准确性较高(MDT讨论)；

3.随访中，结节明显增大或密度变实；

4.患者不伴有影响其生命的其他系统严重基础疾病或其他恶性肿瘤，患者的预期寿命超过5年。

**手术原则与手术切除范围**

1.若病灶位于周边"优势部位"：行肺楔形切除；

2.若病灶位置较深，但仍位于某一肺段内：行肺段切除；

3.病灶位于多个肺段之间或支气管根部：行联合肺段切除或肺叶切除；

4.手术切缘应符合基本肿瘤学原则。

**淋巴结清扫范围**

术中冰冻病理初步诊断为MIA者，无需淋巴结清扫或采样。

**术后辅助治疗**

术后无需放疗、化疗或靶向治疗。如果术中病理为MIA，行楔形切除，术后病理升级为浸润型腺癌，根据浸润型腺癌亚型决定下一步处理方案：如果为微乳头型或实体型，建议再次手术行肺段切除或肺叶切除；如果为贴壁样生长型、乳头型或腺管型，建议随访。

**预后**

完全切除后肿瘤学预后良好，5年生存率可达100%，累积复发率为0%。

**术后随访**

若无明显残余病灶，MIA术后可每年复查一次胸部CT平扫，不必复查头颅磁共振、全身骨扫描或血肿瘤标志物。

## 4 浸润性肺癌

浸润性肺癌即具有手术切除的指征，因此术前的准确判断至关重要。影像学上，非钙化的实性结节若无典型良性结节特征(如错构瘤、胸膜旁结节)，直径大于8 mm或体积大于300 mm3^[25]^，边界存在毛刺征，则恶性可能大^[2]^。部分实性结节如果实性部分直径大于5 mm^[23]^，或者实性面积占比总面积(consolidation-to-tumor ratio)大于25%^[28]^，或者肿瘤纵隔窗消失率(tumor disappearance ratio)小于50%^[39]^，往往提示结节已呈浸润发展。对于pGGN，结节直径大于15 mm、存在支气管充气征、以及CT值 > -472 HU则提示为浸润性肺癌^[40]^。同时，患者年龄(55岁-74岁)、吸烟史( > 30年包、或戒烟年限 < 15年)、既往恶性肿瘤和家族史、肺部合并疾病(慢性阻塞性肺病、肺纤维化)和职业接触史(石棉)等均应纳入结节综合评价^[41]^。

对疑诊浸润性肺癌，术前检查需判断结节的可切除性，主要评估肿瘤临床分期和患者躯体功能^[41]^。前者包括原发肿瘤评估(胸部CT、支气管镜)，纵隔淋巴结评估(纵隔镜、EBUS或EUS-FNA^[42]^)，和远处转移情况[(PET/CT、上腹部超声/CT(包含肾上腺)和头颅磁共振检查]。躯体功能评估主要指患者心肺储备功能、合并症以及血液学检查(血常规、肝肾功能和凝血情况) ^[41]^。美国胸科医师协会(American College of Chest Physicians, ACCP)指南规定若术后第一秒用力呼气量预计值(ppoFEV_1_%)和/或一氧化碳弥散量预计值(ppoDLCO%)小于30%，或者峰值氧耗量(VO_2peak_)小于10 mL/kg/min或小于35%预计值，则为手术高危人群，围术期死亡率较高^[43]^。对于FEV_1_%和DLCO%均大于80%者，往往可耐受全肺切除术^[44]^。

戒烟指导和呼吸功能锻炼应贯穿肺癌手术治疗的始终。ERS/ESTS指南建议术前应至少戒烟2周-4周，否则将增加术后并发症发生的风险^[44]^。围术期有效的呼吸功能锻炼，也可增加手术安全性，同时减少住院天数^[45]^。

选择何种手术方式和途径主要与肿瘤生物学特性、患者因素和手术者经验有关。目前研究已证实微创手术，如电视辅助胸腔镜(video-assisted thoracoscopic surgery, VATS)在早期肺癌治疗的远期预后，包括局部控制率和长期生存率，不亚于传统开胸手术^[46, 47]^；并且在围术期安全性、住院花费和生活质量方面优于开胸手术^[47, 48]^。

手术切除范围主要与肿瘤大小和位置等因素有关，也受患者肺功能和基础疾病的影响。目前NCCN和ACCP指南均指出，解剖性肺叶切除术+淋巴结采样/清扫仍是早期非小细胞肺癌手术切除的标准术式^[41, 49]^。近些年研究发现对于早期肺癌(< 2 cm)，亚肺叶切除术和肺叶切除术在远期生存率上并无统计学差异^[50]^，尤其在亚肺叶切除术联合纵隔淋巴结采样时^[51]^。亚肺叶切除应保证足够的切缘，且切缘直径需大于2 cm或大于肿瘤直径。ACCP指南认为，对于pGGN直径小2 cm，在保证切缘的情况下，也可行亚肺叶切除^[49]^。欧洲临床肿瘤学会(European Society for Medical Oncology, EMSO)指出，结节在PET-CT上呈低摄取可能是亚肺叶切除的良好指征^[52]^。最近一项基于SEER大数据的研究显示，对于 < 1 cm的肿瘤也可行楔形切除术^[53]^。相比以上的研究和指南，英国胸科协会(British Thoracic Society)分析了既往相关文献，认为亚肺叶切除仅适用于pGGN，对于部分实性结节，目前支持亚肺叶切除的证据尚不足^[2]^。但也有研究认为，pGGN和部分实性结节行亚肺叶切除和肺叶切除，在3年无复发率上无显著差异^[54]^。实性结节是否可行亚肺叶切除，仍有待进一步临床研究证实。在组织学类型上，多数研究表明鳞癌则更推荐行标准肺叶切除术^[55]^。对于老年、心肺储备功能较差患者，亚肺叶切除亦是一个较好的选择^[56]^。

对于术中淋巴结处理的方式，是否淋巴结清扫比纵隔淋巴结采样提高肺癌总体生存期，仍存在较多争议^[57]^。对于清扫或采样个数，随机对照试验(ACOSOG Z0030)研究结果认为至少采样12个淋巴结^[58]^，AJCC指南推荐至少采样6个淋巴结，其中需有3站取自纵隔淋巴结(包括第7组)，3站取自肺内淋巴结^[59]^。

浸润性腺癌患者，推荐常规行基因突变检测(推荐常规检测靶标包括*EGFR*、*ALK*、*KRAS*、*ROS1*、*RET*)。目前关于术后辅助治疗，对已行完整切除的Ia期肺癌患者不推荐行辅助治疗^[60]^，Ia期的微乳头型肺癌，复发风险较高，是否需要辅助治疗，尚缺乏证据，目前已有相关临床研究开展。对于Ib期肺癌，如果存在复发的高危因素，如肿瘤细胞低分化(或组织学亚型为微乳头型或实体型)、病理上存在血管或脏层胸膜受侵、淋巴结情况未评估、或者手术切除范围仅为楔形切除，则推荐行辅助化疗^[41]^。对于完整切除的Ⅱ期-Ⅲ期肺癌，推荐术后行全身性辅助治疗，包括化疗或靶向治疗(基因检测突变阳性者) ^[61]^，切缘阳性者需增加切除范围或在术后辅以放疗^[41]^。

在早期肺癌预后方面，腺癌病理亚型是影响预后的主要因素。根据2011年IASLC/ATS/ERS联合推出的腺癌组织学分类^[1]^，将浸润性腺癌分为五大类，包括贴壁型、腺泡型、乳头型、微乳头型和实体型。其中微乳头型和实体型预后最差，腺泡型和乳头型预后次之，贴壁型预后较好^[62, 63]^。浸润性腺癌的处理流程如图 3所示。

**3:**
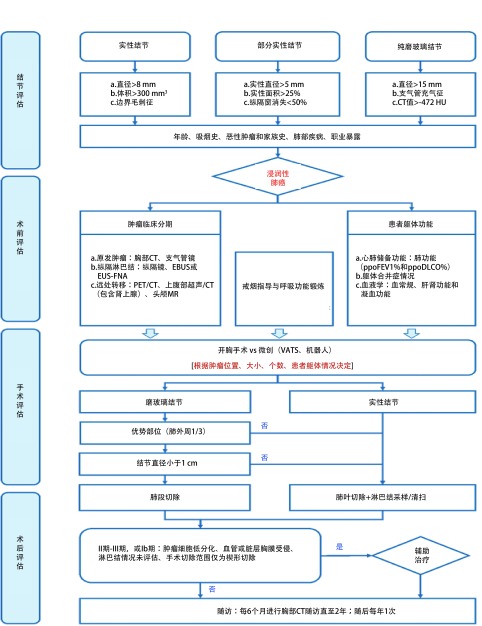
浸润性腺癌的处理流程图 Management of invasive adenocarcinoma

【**肺科共识**——**浸润性腺癌**】

**术前检查**

术前推荐薄层CT平扫，必要时术前行头颅磁共振，全身骨扫描等检查，如果存在明显肿大纵隔淋巴结，行PET/ CT检查，或EBUS，纵隔镜检查排除N2疾病。

**手术原则与手术切除范围**

解剖性肺叶切除术+淋巴结采样/清扫仍是浸润性肺腺癌的标准治疗方式，如果病灶较小的非实性结节(< 1 cm)，或术中冰冻提示贴壁样生长为主型腺癌，可以考虑肺段切除+淋巴结采样/清扫。

**淋巴结清扫范围**

推荐清扫或采样3组6个以上纵隔淋巴结，至少包括第7组淋巴结。

**术后辅助治疗**

根据病理分期以及基因检测结果，决定是否行辅助治疗，以及辅助治疗的策略。

**术后随访**

按肺癌NCCN指南随访。

## 5 肺部多发结节的处理

由于胸部CT影像技术的不断提高，同期多发肺部磨玻璃结节(synchronous multiple ground-glass nodules, SMGN)的检出率也呈上升趋势；研究显示，大约20%-30%的GGN患者，存在肺内多发的GGN病变。目前多数学者认为其更可能是同期多原发肺癌(synchronous multiple primary lung cancers, SMPLC)，而非转移性肺癌^[64]^。其中多原发病理类型为腺癌者约占80%以上^[65]^。

关于同期多原发肺癌的诊断，目前主要依据Martini标准(M -M标准)和ACCP指南。1 9 7 5年Martini和Melamed率先建立了同期多原发肺癌的临床病理诊断标准^[66]^，包括：(a)肿瘤部位不同且相互独立；(b)组织学类型不同；(c)组织学类型相同，但位于不同的肺段、肺叶或双侧肺，起源于不同的原位癌，共同的淋巴引流区域无癌，无肺外转移。随后，在2013年ACCP指南对其做了更新^[67]^，具体为：(a)组织学类型不同，分子遗传学特征不同，或起源于不同的原位癌；(b)组织学类型相同时，肺癌位于不同肺叶，且无N2、N3转移，无远处转移。分子生物学检测对诊断同期多原发肺癌有了很大的提高，如克隆分析(clonality analysis)、杂合性丢失(loss of heterozygosity)等，但也同样面临着挑战，如肿瘤细胞内在异质性^[68]^。

同期多发肺部结节的术前检查，往往需要行PETCT和/或头颅磁共振排除远处转移，并通过胸部CT、支气管镜对纵隔情况进行评估。多发肺部结节的分期，根据最新国际肺癌研究协会(International Association for the Study of Lung Cancer, IASLC)提出的8版分期指南，对已确诊的多原发肺癌患者，应根据每一个肺癌结节分别制定肿瘤-淋巴结-转移(tumor-node-metastasis, TNM)分期。对于CT上表现为多发GGNs的肺癌患者(多数为贴壁型腺癌、微浸润腺癌或原位腺癌)，T分类则根据最高结节的T分期，然后在括号内标注多发GGNs个数(#)或用字母"m"表示。

对同期多发肺部结节的治疗，目前相关高质量的研究较少。一项针对全球范围的调查研究发现，81%的外科医生倾向行手术切除，手术方式以肺叶切除术(针对主要病灶)联合肺段切除术(针对次要病灶)为主^[69]^。有研究结果显示，仅主病灶与患者生存期相关，而是否存在残留结节、残留结节是否增长、有无新发GGNs均与预后无关^[70]^。因此，对于多发GGN，手术切除范围应根据结节具体位置而定，需优先考虑主病灶的切除^[71]^。如果多个GGN处于同一肺叶内，可行多处肺楔形/肺段切除、或者整个肺叶切除；如果多个GGN位于同侧的不同肺叶内，应根据病灶的位置，个体化设计手术方式，可行肺叶/肺段切除联合多处肺段或楔形切除。研究发现，手术中包含楔形或肺段切除术并不影响患者预后，而行全肺切除术患者则预后较差^[72]^。因此，对于SMGN切除所有病灶时，需在符合肿瘤学原则的基础上，尽可能保留肺功能；亚肺叶切除(楔形切或段切)是可行的手术方式，但不推荐行全肺切除术^[73]^。

如果多个GGN位于双侧肺，可同期或分期行肺切除术。2013年ACCP指南规定，若考虑为多原发肺癌，则应尽量做到根治性切除^[67]^。同期或分期的选择，主要取决于患者心肺储备功能，并且与术者及医院经验相关。同期手术可通过一次手术麻醉将病灶全部切除，减少再次手术创伤的应激^[74]^。双侧同期手术增加围术期手术风险，特别是呼吸衰竭的风险。分期手术时，由于前次手术创伤，往往需间隔6周-8周的时间窗，这对于患者是一种精神消耗。如果同期手术安全，应先行手术切除范围较小的一侧，以确保对侧手术的安全实施；如果同期手术存在风险，应先切除主病灶，在情况允许下再行对侧手术^[68]^，一般要求肺的总切除范围不宜超过10个肺段。分期手术时则应先切除主病灶，二期再行对侧手术。双侧浸润性病变行双侧纵隔淋巴结清扫/采样时，应注意神经保护(膈神经和迷走神经)，以免引起双侧膈肌瘫痪或胃瘫。

多原发肺癌预后公认的危险因素包括纵隔淋巴结侵犯和最高肿瘤T分期^[75]^。而多发结节的个数、位置是否位于同侧肺、以及组织病理学是否一致对预后的影响，目前仍有争议^[76]^。关于辅助化疗，目前尚无明确研究证实其能使多原发肺癌患者生存获益^[72]^；因此辅助化疗的选择，仍应根据肿瘤分期，而非结节个数。

总的来说，对于肺部多发GGN疑诊多原发肺癌时，应评估纵隔淋巴结情况(PET/CT、EBUS或纵隔镜)，如果N2淋巴结阳性，则不推荐手术治疗；N2淋巴结阴性时，根据患者病灶分布，心肺功能及体力状况，来决定是切除所有病灶，还是切除主病灶；应根据术者和医院经验选择同期或分期手术，但不推荐行单侧全肺切除术，慎重行同期双侧肺叶切除术。当CT表现多发GGN时，应优先处理主病灶；对于次要GGN病灶，如在同侧，且位于优势部位，可考虑同期手术切除，如在对侧且考虑为AAH或AIS，可密切随访。对于无法完全切除所有病灶的患者，残余病灶应进行密切随访，若随访过程中病灶出现进展，可根据患者情况，考虑再次手术、立体定向放疗(stereotactic body radiation therapy, SBRT)或多学科综合治疗。多发肺磨玻璃结节的处理如图 4所示。

**4:**
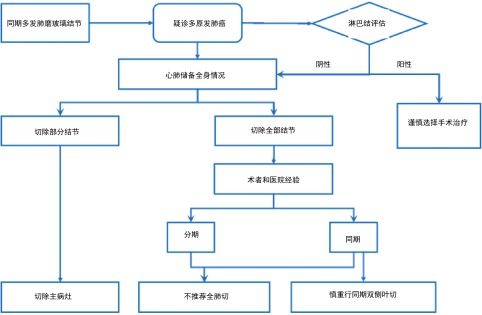
多发肺磨玻璃结节的处理流程图 Management of multiple ground-glass nodules

【**肺科共识**——**多原发磨玻璃结节**】

**术前检查**：术前推荐薄层平扫CT，必要时术前行头颅磁共振，全身骨扫描等检查，如果存在明显肿大纵隔淋巴结，行PET/CT检查，或EBUS，纵隔镜检查排除N2疾病。

**手术原则与手术切除范围**：多原发肺癌的治疗原则为主病灶优先，兼顾次要病灶。多个GGN处于同一肺叶内，可行多处肺楔形切除、肺段切除或肺叶切除，如多个GGN位于同一侧的多个肺叶内，应根据病灶的位置，个体化设计手术方式，行多处肺段切除或楔形切除，符合肿瘤学原则基础上，以尽可能保留肺功能为宜，不推荐行全肺切除术。如多个GGN位于双侧肺内，可同期或分期行双侧VATS肺切除术。双侧手术者，同期双侧肺叶切除需要慎重考虑，肺的总切除范围不宜超过10个肺段。优先处理主病灶；对于次要GGN病灶，如在同侧，且位于优势部位，可考虑同期手术切除，如在对侧且考虑为AAH或AIS，可密切随访。

**淋巴结清扫范围**：推荐清扫或采样3组6个以上纵隔淋巴结，至少包括第7组淋巴结。

**术后辅助治疗**：多原发病灶中的最高分期病灶为是否行辅助治疗之标准，结合肿瘤基因检测结果，决定辅助治疗的策略。主病灶经手术切除后是否残留次要病灶，不作为辅助治疗的选择依据。

**术后随访**：按肺癌NCCN指南随访。

## References

[1] Travis WD, Brambilla E, Noguchi M (2011). International Association for the Study of Lung Cancer/American Thoracic Society/European Respiratory Society International Multidisciplinary Classification of Lung Adenocarcinoma. J Thorac Oncol.

[2] Callister ME, Baldwin DR, Akram AR (2015). British Thoracic Society guidelines for the investigation and management of pulmonary nodules. Thorax.

[3] Naidich DP, Bankier AA, MacMahon H (2013). Recommendations for the management of subsolid pulmonary nodules detected at CT: A statement from the Fleischner Society. Radiology.

[4] Maeshima AM, Tochigi N, Yoshida A (2010). Clinicopathologic analysis of multiple (five or more) atypical adenomatous hyperplasias (AAHs) of the lung: evidence for the AAH-adenocarcinoma sequence. J Thorac Oncol.

[5] Xiang W, Xing Y, Jiang S (2014). Morphological factors differentiating between early lung adenocarcinomas appearing as pure ground-glass nodules measuring </=10 mm on thin-section computed tomography. Cancer Imaging.

[6] Xing Y, Li Z, Jiang S (2015). Analysis of pre-invasive lung adenocarcinoma lesions on thin-section computerized tomography. Clin Respir J.

[7] Lee SM, Goo JM, Lee KH (2015). CT findings of minimally invasive adenocarcinoma (MIA) of the lung and comparison of solid portion measurement methods at CT in 52 patients. Eur Radiol.

[8] Zhang Y, Qiang JW, Ye JD (2014). High resolution CT in differentiating minimally invasive component in early lung adenocarcinoma. Lung Cancer.

[9] MacMahon H, Naidich DP, Goo JM (2017). Guidelines for management of incidental pulmonary nodules detected on CT images: From the Fleischner Society 2017. Radiology.

[b10] 10Japanese Society for CT Screening. Guidelines for the management of pulmonary nodules detected by low-dose CT Lung Cancer Screening Version 3 2013. Available at: http://www.jscts.org/pdf/guideline/gls3rd_english130621.pdf. Accessed date, 20 Feb, 2018.

[11] Gould MK, Donington J, Lynch WR (2013). Evaluation of individuals with pulmonary nodules: when is it lung cancer? Diagnosis and management of lung cancer, 3rd ed: American College of Chest Physicians evidence-based clinical practice guidelines. Chest.

[b12] 12NCCN Clinical Practice Guidelines in Oncology. Lung Cancer Screening Version 2. 2018. 2018. Available at: https://www.nccn.org/professionals/physician_gls/pdf/lung_screening.pdf.

[13] Cho S, Yang H, Kim K (2013). Pathology and prognosis of persistent stable pure ground-glass opacity nodules after surgical resection. Ann Thorac Surg.

[14] Eguchi T, Kondo R, Kawakami S (2014). Computed tomography attenuation predicts the growth of pure ground-glass nodules. Lung Cancer.

[15] Sim HJ, Choi SH, Chae EJ (2014). Surgical management of pulmonary adenocarcinoma presenting as a pure ground-glass nodule. Eur J Cardiothorac Surg.

[16] Cho JH, Choi YS, Kim J (2015). Long-term outcomes of wedge resection for pulmonary ground-glass opacity nodules. Ann Thorac Surg.

[17] Zhang Y, Zhang Y, Chen S (2015). Is bronchoscopy necessary in the preoperative workup of a solitary pulmonary nodule?. J Thorac Cardiovasc Surg.

[18] Cho H, Lee HY, Kim J (2015). Pure ground glass nodular adenocarcinomas: Are preoperative positron emission tomography/computed tomography and brain magnetic resonance imaging useful or necessary?. J Thorac Cardiovasc Surg.

[19] Chang B, Hwang JH, Choi YH (2013). Natural history of pure ground-glass opacity lung nodules detected by low-dose CT scan. Chest.

[20] van den Bergh KA, Essink-Bot ML, Borsboom GJ (2010). Short-term health-related quality of life consequences in a lung cancer CT screening trial (NELSON). Br J Cancer.

[21] Lee SM, Goo JM, Lee KH (2015). CT findings of minimally invasive adenocarcinoma (MIA) of the lung and comparison of solid portion measurement methods at CT in 52 patients. Eur Radiol.

[22] Lim HJ, Ahn S, Lee KS (2013). Persistent pure ground-glass opacity lung nodules >/= 10 mm in diameter at CT scan: histopathologic comparisons and prognostic implications. Chest.

[23] Cohen JG, Reymond E, Lederlin M (2015). Differentiating pre-and minimally invasive from invasive adenocarcinoma using CT-features in persistent pulmonary part-solid nodules in Caucasian patients. Eur J Radiol.

[24] Wilshire CL, Louie BE, Manning KA (2015). Radiologic evaluation of small lepidic adenocarcinomas to guide decision making in surgical resection. Ann Thorac Surg.

[25] Suzuki K, Koike T, Asakawa T (2011). A prospective radiological study of thin-section computed tomography to predict pathological noninvasiveness in peripheral clinical Ia lung cancer (Japan Clinical Oncology Group 0201). J Thorac Oncol.

[26] Lee KH, Goo JM, Park SJ (2014). Correlation between the size of the solid component on thin-section CT and the invasive component on pathology in small lung adenocarcinomas manifesting as ground-glass nodules. J Thorac Oncol.

[27] Nakamura K, Saji H, Nakajima R (2010). A phase Ⅲ randomized trial of lobectomy versus limited resection for small-sized peripheral non-small cell lung cancer (JCOG0802/WJOG4607L). Jpn J Clin Oncol.

[28] Suzuki K, Watanabe S, Mizusawa J (2015). Predictors of non-neoplastic lesions in lung tumours showing ground-glass opacity on thin-section computed tomography based on a multi-institutional prospective studydagger. Interact Cardiovasc Thorac Surg.

[b29] 29Grills IS, Fitch Dl Fau -Goldstein NS, Goldstein Ns Fau -Yan D, *et al*. Clinicopathologic analysis of microscopic extension in lung adenocarcinoma: defining clinical target volume for radiotherapy (0360-3016 (Print)). doi: 10.1016/j.ijrobp.2007.03.023

[30] Higashi K, Ueda Y, Seki H (1998). Fluorine-18-FDG PET imaging is negative in bronchioloalveolar lung carcinoma. J Nucl Med.

[31] Izumo T, Sasada S, Chavez C (2015). Radial endobronchial ultrasound images for ground-glass opacity pulmonary lesions. Eur Respir J.

[32] Fan J, Wang L, Jiang GN (2012). Sublobectomy versus lobectomy for stage Ⅰ non-small-cell lung cancer, a meta-analysis of published studies. Ann Surg Oncol.

[33] Okada M, Koike T, Higashiyama M (2006). Radical sublobar resection for small-sized non-small cell lung cancer: a multicenter study. J Thorac Cardiovasc Surg.

[34] Liu S, Wang R, Zhang Y (2016). Precise diagnosis of intraoperative frozen section is an effective method to guide resection strategy for peripheral small-sized lung adenocarcinoma. J Clin Oncol.

[35] He P, Yao G, Guan Y (2016). Diagnosis of lung adenocarcinoma in situ and minimally invasive adenocarcinoma from intraoperative frozen sections: an analysis of 136 cases. J Clin Pathol.

[36] Luis HA, Zhou Y, Sihoe AD (2017). Survival is not compromised in patients with invasive adenocarcinoma found in GGOs receiving sublobar resection due to intraoperative frozen section ambiguity: A prospensity score matched analysis, 2017. 25th European Conference on General Thoracic Surgery.

[37] Kadota K, Villena-Vargas J, Yoshizawa A (2014). Prognostic significance of adenocarcinoma in situ, minimally invasive adenocarcinoma, and nonmucinous lepidic predominant invasive adenocarcinoma of the lung in patients with stage Ⅰ disease. Am J Surg Pathol.

[38] Eguchi T, Kadota K, Park BJ (2014). The new IASLC-ATS-ERS lung adenocarcinoma classification: what the surgeon should know. Semin Thorac Cardiovasc Surg.

[39] Shimada Y, Yoshida J, Hishida T (2012). Predictive factors of pathologically proven noninvasive tumor characteristics in T1aN0M0 peripheral non-small cell lung cancer. Chest.

[40] Lee HY, Choi YL, Lee KS (2014). Pure ground-glass opacity neoplastic lung nodules: histopathology, imaging, and management. AJR Am J Roentgenol.

[b41] 41NCCN Clinical Practice Guidelines Oncology. Non-Small Cell Lung Cancer. Version 9. Available at: https://www.nccn.org/professionals/physician_gls/pdf/nscl.pdf.

[42] De Leyn P, Dooms C, Kuzdzal J (2014). Revised ESTS guidelines for preoperative mediastinal lymph node staging for non-small-cell lung cancer. Eur J Cardiothorac Surg.

[43] Brunelli A, Kim AW, Berger KI (2013). Physiologic evaluation of the patient with lung cancer being considered for resectional surgery: Diagnosis and management of lung cancer, 3rd ed: American College of Chest Physicians evidence-based clinical practice guidelines. Chest.

[44] Brunelli A, Charloux A, Bolliger CT (2009). ERS/ESTS clinical guidelines on fitness for radical therapy in lung cancer patients (surgery and chemo-radiotherapy). Eur Respir J.

[45] Bade BC, Thomas DD, Scott JB (2015). Increasing physical activity and exercise in lung cancer: reviewing safety, benefits, and application. J Thorac Oncol.

[46] Whitson BA, Groth SS, Duval SJ (2008). Surgery for early-stage non-small cell lung cancer: a systematic review of the video-assisted thoracoscopic surgery versus thoracotomy approaches to lobectomy. Ann Thorac Surg.

[47] Klapper J, D'Amico TA (2015). VATS versus open surgery for lung cancer resection: moving toward a minimally invasive approach. J Natl Compr Canc Netw.

[48] Bendixen M, Jorgensen OD, Kronborg C (2016). Postoperative pain and quality of life after lobectomy via video-assisted thoracoscopic surgery or anterolateral thoracotomy for early stage lung cancer: a randomised controlled trial. Lancet Oncol.

[49] Howington JA, Blum MG, Chang AC (2013). Treatment of stage Ⅰ and Ⅱ non-small cell lung cancer: Diagnosis and management of lung cancer, 3rd ed: American College of Chest Physicians evidence-based clinical practice guidelines. Chest.

[50] Altorki NK, Yip R, Hanaoka T (2014). Sublobar resection is equivalent to lobectomy for clinical stage 1a lung cancer in solid nodules. J Thorac Cardiovasc Surg.

[51] Cox ML, Yang CJ, Speicher PJ (2017). The role of extent of surgical resection and lymph node assessment for clinical stage Ⅰ pulmonary lepidic adenocarcinoma: An analysis of 1, 991 patients. J Thorac Oncol.

[52] Postmus PE, Kerr KM, Oudkerk M (2017). Early and locally advanced non-small-cell lung cancer (NSCLC): ESMO Clinical Practice Guidelines for diagnosis, treatment and follow-up. Ann Oncol.

[53] Dai C, Shen J, Ren Y (2016). Choice of Surgical Procedure for Patients With Non-Small-Cell Lung Cancer </= 1 cm or > 1 to 2 cm Among Lobectomy, Segmentectomy, and Wedge Resection: A Population-Based Study. J Clin Oncol.

[54] Tsutani Y, Miyata Y, Nakayama H (2014). Appropriate sublobar resection choice for ground glass opacity-dominant clinical stage Ⅰa lung adenocarcinoma: wedge resection or segmentectomy. Chest.

[55] Veluswamy RR, Ezer N, Mhango G (2015). Limited resection versus lobectomy for older patients with early-stage lung cancer: Impact of histology. J Clin Oncol.

[56] Sihoe AD, Van Schil P (2014). Non-small cell lung cancer: when to offer sublobar resection. Lung Cancer.

[57] Darling GE, Allen MS, Decker PA (2011). Number of lymph nodes harvested from a mediastinal lymphadenectomy: results of the randomized, prospective American College of Surgeons Oncology Group Z0030 trial. Chest.

[58] Darling GE, Allen MS, Decker PA (2011). Randomized trial of mediastinal lymph node sampling versus complete lymphadenectomy during pulmonary resection in the patient with N0 or N1 (less than hilar) non-small cell carcinoma: results of the American College of Surgery Oncology Group Z0030 Trial. J Thorac Cardiovasc Surg.

[59] Detterbeck FC, Postmus PE, Tanoue LT (2013). The stage classification of lung cancer: Diagnosis and management of lung cancer, 3rd ed: American College of Chest Physicians evidence-based clinical practice guidelines. Chest.

[60] Kris MG, Gaspar LE, Chaft JE (2017). Adjuvant systemic therapy and adjuvant radiation therapy for stage Ⅰ to Ⅲ a completely resected non-Small-cell lung cancers: American Society of Clinical Oncology/Cancer Care Ontario Clinical Practice Guideline Update. J Clin Oncol.

[61] Bradbury P, Sivajohanathan D, Chan A (2017). Postoperative adjuvant systemic therapy in completely resected non-small-cell lung cancer: A systematic review. Clin Lung Cancer.

[62] Warth A, Muley T, Meister M (2012). The novel histologic International Association for the Study of Lung Cancer/American Thoracic Society/European Respiratory Society classification system of lung adenocarcinoma is a stage-independent predictor of survival. J Clin Oncol.

[63] Hung JJ, Yeh YC, Jeng WJ (2014). Predictive value of the international association for the study of lung cancer/American Thoracic Society/European Respiratory Society classification of lung adenocarcinoma in tumor recurrence and patient survival. J Clin Oncol.

[64] Detterbeck FC, Chansky K, Groome P (2016). The IASLC lung cancer staging project: Methodology and validation used in the development of proposals for revision of the stage classification of NSCLC in the forthcoming (Eighth) edition of the TNM classification of lung cancer. J Thorac Oncol.

[65] Yu YC, Hsu PK, Yeh YC (2013). Surgical results of synchronous multiple primary lung cancers: similar to the stage-matched solitary primary lung cancers?. Ann Thorac Surg.

[66] Martini N, Melamed MR (1975). Multiple primary lung cancers. J Thorac Cardiovasc Surg.

[67] Kozower BD, Larner JM, Detterbeck FC (2013). Special treatment issues in non-small cell lung cancer: Diagnosis and management of lung cancer, 3rd ed: American College of Chest Physicians evidence-based clinical practice guidelines. Chest.

[68] Loukeri AA, Kampolis CF, Ntokou A (2015). Metachronous and synchronous primary lung cancers: diagnostic aspects, surgical treatment, and prognosis. Clin Lung Cancer.

[69] Leventakos K, Peikert T, Midthun DE (2017). Management of multifocal lung cancer: Results of a survey. J Thorac Oncol.

[70] Shimada Y, Saji H, Otani K (2015). Survival of a surgical series of lung cancer patients with synchronous multiple ground-glass opacities, and the management of their residual lesions. Lung Cancer.

[71] Gao RW, Berry MF, Kunder CA (2017). Survival and risk factors for progression after resection of the dominant tumor in multifocal, lepidic-type pulmonary adenocarcinoma. J Thorac Cardiovasc Surg.

[72] Kocaturk CI, Gunluoglu MZ, Cansever L (2011). Survival and prognostic factors in surgically resected synchronous multiple primary lung cancers. Eur J Cardiothorac Surg.

[73] Liu M, He W, Yang J (2016). Surgical treatment of synchronous multiple primary lung cancers: a retrospective analysis of 122 patients. J Thorac Dis.

[74] Mun M, Kohno T (2007). Single-stage surgical treatment of synchronous bilateral multiple lung cancers. Ann Thorac Surg.

[75] Tanvetyanon T, Finley DJ, Fabian T (2013). Prognostic factors for survival after complete resections of synchronous lung cancers in multiple lobes: pooled analysis based on individual patient data. Ann Oncol.

[76] Fabian T, Bryant AS, Mouhlas AL (2011). Survival after resection of synchronous non-small cell lung cancer. J Thorac Cardiovasc Surg.

